# The potential of peritoneal dialysis as a cost-effective and sustainable treatment option for chronic kidney disease: A perspective from a resource-limited country like Pakistan

**DOI:** 10.12669/pjms.39.6.8560

**Published:** 2023

**Authors:** Rahima Azam, Maryam Raza

**Affiliations:** 1 Rahima Azam; 2 Maryam Raza

Chronic Kidney Disease or CKD has been recognized as a leading public health problem worldwide. The incidence of the disease has been increasing, with a significant number of these patients progressing to end stage renal disease (ESRD) every year. The treatment modality of ESRD includes renal transplant or dialysis. The ideal treatment is a renal transplant, but that too has its setbacks including being ineligible for transplantation, the high cost associated with it and the non-availability of organs for donation.^1^ Therefore, most people end up opting for dialysis, which can be done by either of the two methods: hemodialysis (HD) or peritoneal dialysis (PD). In developing countries, the most used technique is HD, PD is not being readily used. PD is only available in 29% of low-income and 68% of lower-middle income countries compared with 97% and 100% in upper-middle and high-income countries.^2^

The efficacy of HD and PD is equal. In fact, PD can prove to be more beneficial in resource limited countries such as ours. Firstly, HD must be done in specialized dialysis centers, three times per week with each session lasting four hours, whereas PD can be done in the comfort of your home, with the time duration being much shorter, and can also be done at nighttime while the patient is sleeping. PD also allows for less restriction in diet and fluid intake in comparison to HD. This allows for patients to have a better quality of life, have a sense of autonomy, have greater flexibility, provide better cosmesis as compared to HD vascular access sites, not having to be pricked repeatedly for cannulation for HD, and most importantly, avoidance of regular hospital visits for patients and their care takers.^3^ This allows them to continue their day-to-day lives and jobs regularly and reduces time and cost associated with travel to and from the hospital.

This is especially important in a resource-limited setting such as ours. PD can also be more beneficial for the environmental and economic situation of Pakistan as it has proven to be beneficial in terms of solute clearance, consumption of water and electricity, and production of waste products. The cost for PD is also much less than HD. Per month the cost for HD is Rs. 54,000 as compared to Rs.30-35,000 for PD.^4^

To conclude developing countries like Pakistan, which have limited availability of resources and where the economy is already collapsing, PD is a much better option than HD, as it can not only help the people at an individual level but can also benefit at a national level, by reducing the economic burden and helping in conserving the limited resources that we have. The Government of Pakistan, NGOs and the private sector should work together in introducing and making PD an option to consider for people undergoing treatment for ESRD, for the betterment of the country and its people.
